# Knowledge of human papillomavirus and self-sampling, including vaccination practices among female students in Free State, South Africa

**DOI:** 10.1007/s10552-025-02049-5

**Published:** 2025-08-23

**Authors:** Teboho Amelia Tiiti, Omololu Aluko, Claire Barrett

**Affiliations:** 1 Department of Internal Medicine, Faculty of Health Sciences, University of the Free State, Bloemfontein, 9300 South Africa; 2 Department of Biostatistics, School of Clinical Medicine, Faculty of Health Sciences, University of the Free State, Bloemfontein, 9300 South Africa; 3 School of Clinical Medicine, Faculty of Health Sciences, University of the Free State, Bloemfontein, 9300 South Africa

**Keywords:** Human papillomavirus, Vaccination, Self-sampling, Knowledge, Public health

## Abstract

**Background and Aim:**

Human papillomavirus (HPV)-related cancers, especially cervical cancer, remain highly prevalent in low- and middle-income countries, despite the availability of preventive measures such as vaccination and self-sampling screening, due to limited HPV awareness. The study aimed to assess the knowledge of HPV, HPV vaccination practices, and HPV self-sampling awareness and perceptions among female students at the University of the Free State in Bloemfontein, South Africa.

**Methods:**

Data were collected from female university students through a self-administered questionnaire distributed via the secure web-based platform Research Electronic Data Capture (REDCap). HPV infection and vaccination knowledge were measured using a self-administered questionnaire. Knowledge was assessed by assigning one (1) point for each correct answer, while incorrect or “don't know” responses received a score of zero (0). A knowledge score above 75% was categorized as “good knowledge.” The data were analyzed using SAS version 9.4.

**Results:**

The study included 381 participants with a median age (interquartile range, IQR) of 23.0 (20–26) years. The findings showed that while 40.9% of participants had good knowledge of HPV infection, only 9.7% demonstrated good knowledge of HPV vaccination, and 13.3% had good overall knowledge. Only 13.4% of participants reported having received the HPV vaccine, while 19.1% expressed unwillingness to receive the vaccine. Barriers to vaccine uptake included lack of information about the HPV vaccine (46.0%) and safety concerns (46.0%). The majority (78.2%) were unaware of HPV self-sampling. Having heard about HPV self-sampling was predictive for HPV knowledge (OR: 2.684, 95% CI: 1.389–5.188, *p* = 0.003).

**Conclusion:**

These findings suggest that while some participants are informed about HPV infection, the majority are not well-informed about HPV vaccination and are unaware of HPV self-sampling. Additionally, barriers to HPV vaccination persist. Targeted educational interventions are needed to address awareness and knowledge gaps and vaccine hesitancy. These interventions could significantly improve HPV and cervical cancer prevention outcomes.

**Supplementary Information:**

The online version contains supplementary material available at 10.1007/s10552-025-02049-5.

## Introduction

Human papillomavirus (HPV) is one of the most prevalent sexually transmitted infections (STIs), and it has significant health implications, including the development of cervical cancer [1]. Cervical cancer is the leading cause of cancer deaths among women in South Africa [2]. In 2020, an estimated 10,702 new cases of cervical cancer were diagnosed in South Africa, leading to 5,870 deaths [2]. Globally, an estimated 80% of sexually active women contract HPV during their lifetime [3]. In most cases, the host’s immune system clears the virus naturally within 1–2 years [4]. If the immune system does not clear the virus, it can persist latently [5].

HPV infections are classified as either high-risk (hr-) or low-risk (lr-) based on their oncogenic potential [6]. Persistent infection with high-risk HPV types, particularly HPV16 and HPV18, can increase the risk of developing precancerous lesions, which can eventually progress to invasive cancers [7]. In South Africa, the burden of HPV16 and HPV18 infections varies by cytology, ranging from 3.2% among women with normal cytology (general population) to 64.2% among those with cervical cancer [2]. In Africa, young women have been shown to have a higher prevalence of HPV [8–10].

HPV is primarily prevented through prophylactic vaccination [7]. In 2008, South Africa approved the bivalent (Cervarix®, targeting HPV16 and HPV18) and quadrivalent (Gardasil®, targeting HPV6, HPV11, HPV16, and HPV18) HPV vaccines. The school-based vaccination program launched in 2014 targets grade 4 girls aged nine and older in public schools [11]. As part of global efforts to eliminate cervical cancer, the World Health Assembly adopted the Global Strategy for Cervical Cancer Elimination in 2020 [12].

Secondary prevention of HPV includes HPV testing, which is more sensitive for detecting cervical intraepithelial neoplasia grade 2 or worse (CIN2 +) [13]. Fortunately, HPV testing allows for self-collection, where women collect cervicovaginal samples using a swab or brush, which is then tested for hr-HPV [14]. HPV self-collection has emerged as a potential solution to increase screening rates, particularly for under-screened or unscreened women [15]. Despite South African cervical cancer policy recommendations, HPV self-sampling is not yet widely implemented in screening programs [16], highlighting the importance of assessing women’s awareness and perceptions of this method. However, the policy provides for three free cervical cancer screening tests, offered at ten-year intervals, for all asymptomatic women over the age of 30 who attend public sector health services [16].

Despite the availability of prevention strategies, gaps in HPV awareness and knowledge persist, particularly in LMICs, including South Africa, where cervical cancer rates remain high. Studies have shown a lack of knowledge about HPV infection and HPV vaccination among university students in South Africa [15, 17–19]. Furthermore, there is limited research on vaccine uptake and the awareness and perceptions toward HPV self-sampling among this population in South Africa. This study aimed to assess the knowledge of HPV, HPV vaccination practices, and HPV self-sampling awareness and perceptions among female students at the University of the Free State (UFS) in South Africa. The study seeks to identify gaps in understanding, as well as practices related to the HPV vaccine.

## Methods

### Study design and population

This was an online cross-sectional study conducted among female students at the UFS, Bloemfontein Campus, South Africa. Participants were recruited via email using a convenience sampling method, a non-probability sampling technique. Inclusion criteria were as follows: (i) female students, (ii) aged 18 years and older, and (iii) enrolled at the Bloemfontein Campus. Study data were collected and managed using the Research Electronic Data Capture (REDCap) tool hosted by the UFS [20, 21]. Data collection took place from May 2024 to November 2024 through an electronic self-administered questionnaire.

### Survey instrument

HPV infection and vaccination knowledge were measured using a self-administered questionnaire developed by the authors for this study. The questionnaire was developed in English and comprised five sections: demographics, HPV infection knowledge, HPV vaccination knowledge, vaccination practices, awareness, and perceptions of HPV self-sampling. The first section included demographic data (age, marital status, educational level), sexual practices (sexual activity, contraceptive use, number of sexual partners, history of sexually transmitted infections (STIs), recent healthcare visits), and family history of cervical cancer. The second and third sections consisted of ten true/false/don’t know questions, each about HPV infection and HPV vaccination, respectively. The fourth section addressed vaccination practices, and the fifth section focused on HPV self-sampling awareness and perceptions. Participants were asked to download and read an attached document on the survey detailing HPV self-sampling before answering questions on awareness and any potential concerns toward self-sampling. Since HPV self-sampling is a novel method, the document served as an educational tool, ensuring that participants had a baseline understanding of HPV self-sampling before answering questions (Supplementary file 1).

### Pilot study

A pilot study was conducted with five students to assess the clarity of the questionnaire. Participants were asked whether the questions were understandable and to identify any unclear items. A biostatistician reviewed the pilot data and confirmed the questionnaire’s clarity and effectiveness. No changes were made, and the pilot data were included in the final study.

### Participant recruitment

Participants were recruited using a research information document, which included a link to the electronic survey. The UFS Department of Communication and Marketing facilitated this process. The document included the study’s aim, design, eligibility criteria, researcher information, and instructions on accessing the survey. Additionally, the UFS Facebook page was used to improve response rates and raise awareness about the study.

### Post-survey feedback

After completing the survey, participants received a document with the correct answers to the HPV knowledge-based questions. This document was included in the survey feedback process to enhance participants’ understanding of HPV infection and vaccination by providing accurate information immediately upon survey completion. This feedback mechanism aimed to address potential knowledge gaps and strengthen the educational component of the study.

### Outcome measures

The primary outcome was participants’ knowledge scores, calculated based on correct responses to 20 knowledge-based questions. Correct answers were assigned a point ‘1,’ while incorrect and ‘don’t know’ responses were scored as ‘0.’ Missing answers were grouped with ‘don’t know’ responses. Participants’ knowledge levels were categorized into three groups: < 50% (poor knowledge), 50–75% (moderate knowledge), and > 75% (good knowledge) [22].

### Ethics approval

Ethics approval for this study was obtained from the UFS Health Sciences Research Ethics Committee (HSD2023/2368/2702). Institutional gatekeepers also granted permission to conduct the research. Participation was voluntary, and implicit consent was inferred upon survey completion, as outlined by the following statement: “Please note that by completing this survey, you are voluntarily agreeing to participate in this research study.”

### Sample size

The sample size was calculated using Epi Info™ version 7 (Centers for Disease Control and Prevention). The calculation was based on a population size of 29,684 students at the UFS Bloemfontein Campus, with a 95% confidence interval (CI), a 5% margin of error, and an expected response frequency of 50%, yielding a sample size of 379.

## Data analysis

Participants who did not fully complete the survey were excluded from the analysis to ensure data quality and integrity. The internal consistency of the knowledge-based questions was assessed using Cronbach’s analysis. Frequencies (*n*) and percentages (%) were calculated to summarize categorical data, while descriptive statistics, median, and interquartile range (IQR) were used for continuous variables such as age. Factors associated with HPV knowledge were examined using logistic regression analysis. Both univariate and multivariable regression models were employed to calculate odds ratios (ORs) and adjusted odds ratios (AORs) with 95% confidence intervals (CIs). A backward selection model was used, and variables with *p* > 0.2 were excluded. The rationale for excluding variables with *p* > 0.2 is to remove non-significant variables that have no association with the outcome. This procedure reduces overfitting and improves the model’s interpretability. The significant variables were adjusted for in the multivariable model. Non-significant variables of interest were included in the model. In this case, only one variable was significant, but two variables of interest were included. A two-sided *p* value of less than 0.05 was considered statistically significant. Data were analyzed using SAS version 9.4 (SAS Institute Inc., Cary, NC, USA).

For the open-ended question, “Do you have any potential concerns regarding HPV self-sampling?” the responses were categorized as follows:

- **Accuracy and quality of samples**: Concerns about the accuracy, reliability, and validity of the self-sampling process, including comparisons with samples taken by healthcare professionals.
- **Procedure concerns**: Issues related to safety, discomfort, and specific concerns for individuals with unique conditions (e.g., intact hymen).
- **Education and information**: The need for adequate education and information on HPV self-sampling.
- **Cost and accessibility**: Concerns related to the financial costs and accessibility of self-sampling.
- **Health impact**: Questions about the overall health implications of self-sampling.

## Results

### Questionnaire internal consistency

Cronbach’s alpha, a measure of internal consistency, was used to assess the reliability of the questionnaire. The survey instrument measuring participants’ knowledge of human papillomavirus (HPV) demonstrated good internal consistency with a Cronbach’s alpha coefficient of 0.860 (Supplementary Table 1).

### Characteristics of Participants

Of the 389 survey responses received, eight were excluded from the analysis due to incompleteness or being under the age of 18. Characteristics of the sample show that most (79.3%) of the participants were aged 18–26 years, with a median (interquartile range, IQR) age of 23.0 (20–26) years. The majority (56.2%) were undergraduates. Of the 73.8% participants in sexual relationships, 72.2% used contraceptives, mainly condoms (36.0%). Seventy-five percent (75.1%) of the participants reported having one sexual partner, and 56.7% of the participants visited a healthcare facility in the past 6 months (Table 1).

**Table 1:** Characteristics of female students at the University of the Free State

Variable		*n* (%)
Age		
Median: 23.0; IQR: 20–26 (*n* = 381)	18–26	302 (79.3%)
	27–45	71 (18.6%)
	≥ 46	8 (2.1%)
Educational level (*n* = 381)	Undergraduate	214 (56.2%)
	Postgraduate	167 (43.8%)
Postgraduate level (*n* = 167)	Honours	79 (47.3%)
	Masters	68 (40.7%)
	Doctorate	9 (5.4%)
	Missing	11 (6.6%)
Faculty (*n* = 381)	Health Sciences	125 (32.8%)
	Economics and Management Sciences	68 (17.9%)
	Education	38 (10.0%)
	Law	18 (4.7%)
	Natural and Agricultural Sciences	66 (17.3%)
	Humanities	56 (14.7%)
	Theology and Religion	10 (2.6%)
In a sexual relationship (*n* = 381)	Yes	281 (73.8%)
	No	100 (26.2%)
Contraceptive use (*n* = 281)	Yes	203 (72.2%)
	No	61 (21.7%)
	Missing	17 (6.1%)
Type of contraceptives currently used (*n* = 203) (multiple choices allowed)	Condoms	73 (36.0%)
	Oral contraceptives	58 (28.6%)
	Intrauterine devices (IUDs)	19 (9.4%)
	Injection	58 (28.6%)
	Implant	6 (3.0%)
	Other^a^	2 (1.0%)
Number of current sexual partners (*n* = 281)	1	211 (75.1%)
	2	50 (17.8%)
	3	4 (1.4%)
	Missing	16 (5.7%)
Visited a healthcare facility in the past 6 months (*n* = 381)	Yes	216 (56.7%)
	No	165 (43.3%)
History of STIs in the past six months? (*n* = 381)	Yes	20 (5.2%)
	No	360 (94.5%)
	Missing	1 (0.3%)
Family history of cervical cancer (*n* = 381)	Yes	37 (9.7%)
	No	344 (90.3%)

*n* = frequency

% = percentage

IQR: interquartile range

^a^Includes sterilization and contraceptive patch

### Participants’ knowledge of HPV infection and vaccination

Tables 2 and 3 represent participants’ knowledge of human papillomavirus (HPV) infection and HPV vaccination, respectively. The results show that most of the participants identified HPV as sexually transmitted (92.7%) and high-risk (hr-) HPV as a cause of cervical cancer (89.5%). Only 41.5% recognized that the immune system can clear HPV, and a minority (34.6%) knew that HPV cannot be cured. Furthermore, the majority (90.3%) of the participants correctly answered that there is a vaccine against HPV, and 19.4% answered that HPV vaccination is not the most effective secondary prevention of HPV. Only 43.8% of the participants correctly answered that the HPV vaccine cannot eliminate pre-existing HPV infections, and 62.7% knew that vaccinated women should still be screened. About sixty-seven percent (62.7%) of the participants were aware of the school-based vaccination program in South Africa, and 63.8% correctly answered that the vaccine is administered to girls aged 9 to 12 years, and 3.1% knew of the Cervical Cancer Elimination “90–70–90” targets to fully vaccinate girls by the age of 15 and not 12.

**Table 2:** Knowledge about human papillomavirus (HPV) infection among female students at the University of the Free State, *n* = 381

Variable		*n* (%)
HPV is sexually transmitted	True^a^	353 (92.7%)
	False	8 (2.1%)
	Don’t know	20 (5.2%)
High-risk HPV causes cervical cancer	True^a^	341(89.5%)
	False	4 (1.0%)
	Don’t know	36 (9.5%)
HPV does not cause genital warts	True	51 (13.4%)
	False^a^	272 (71.4%)
	Don’t know	58 (15.2%)
HPV does not usually cause any symptoms	True^a^	226 (59.3%)
	False	80 (21.0%)
	Don’t know	75(19.7%)
HPV can be eliminated/cleared spontaneously by our immune system	True^a^	158 (41.5%)
	False	68 (17.8%)
	Don’t know	155 (40.7%)
Condoms protect against HPV infection	True^a^	337 (88.5%)
	False	16 (4.2%)
	Don’t know	28 (7.3%)
Low-risk HPV increases the risk of cervical cancer	True	55 (14.4%)
	False^a^	255 (66.9%)
	Don’t know	71 (18.6%)
HPV 16 and HPV 18 are low-risk HPV types	True	53 (13.9%)
	False^a^	208 (54.6%)
	Don’t know	120 (31.5%)
HPV can be cured	True	91 (23.9%)
	False^a^	132 (34.6%)
	Don’t know	158 (41.5%)
HPV infection is higher in HIV-infected women	True^a^	193 (50.7%)
	False	12 (3.1%)
	Don’t know	176 (46.2%)

*n* = frequency

% = percentage

^a^Correct response

**Table 3:** Knowledge about human papillomavirus (HPV) vaccination among female students at the University of the Free State, *n* = 381

Variable		*n* (%)
There is a vaccine against HPV	True^a^	344 (90.3%)
	False	3 (0.8%)
	Don’t know	34 (8.9%)
HPV vaccination is the most effective secondary prevention of HPV	True	253 (66.4%)
	False^a^	74 (19.4%)
	Don’t know	54 (14.2%)
The HPV vaccine protects against genital warts	True^a^	264 (69.3%)
	False	29 (7.6%)
	Don’t know	88 (23.1%)
The HPV vaccine helps to eliminate an HPV infection that already exists	True	100 (26.3%)
	False^a^	167 (43.8%)
	Don’t know	114 (29.9%)
It is too late to get HPV vaccinated after the first sexual intercourse	True	51 (13.4%)
	False^a^	241 (63.2%)
	Don’t know	89 (23.4%)
Cervical screening is not recommended among vaccinated women	True	44 (11.6%)
	False^a^	239 (62.7%)
	Don’t know	98 (25.7%)
South Africa has implemented a nationwide school-based HPV vaccination program	True^a^	254 (66.7%)
	False	10 (2.6%)
	Don’t know	117 (30.7%)
The HPV vaccine is administered to girls between the ages of 9 and 12	True^a^	243 (63.8%)
	False	16 (4.2%)
	Don’t know	122 (32.0%)
The HPV vaccine is administered in four doses, about six months apart, starting at age nine years	True	142 (37.3%)
	False^a^	71 (19.6%)
	Don’t know	168 (44.1%)
According to the WHO Global Strategy for the Elimination of cervical cancer, 90% of girls should be fully vaccinated with the HPV vaccine by the age of 12	True	169 (44.4%)
	False^a^	12 (3.1%)
	Don’t know	200 (52.5%)

*n* = frequency

% = percentage

^a^Correct response

### Participants’ overall knowledge scores of HPV infection and vaccination

**Fig. 1:**
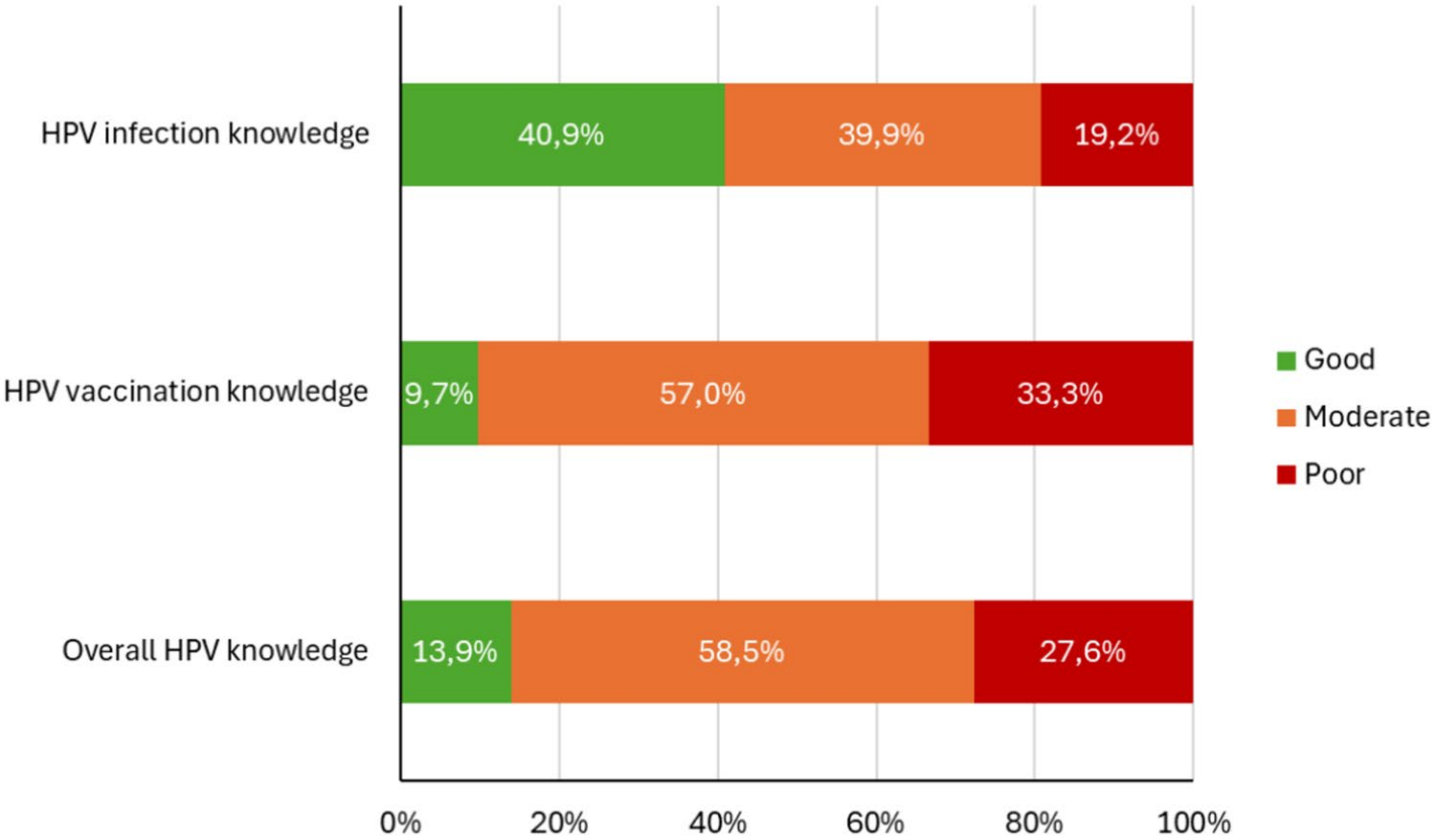
Human papillomavirus (HPV) knowledge scores among female students at the University of the Free State, *n* = 381

Most (40.9%) of the participants had good knowledge of HPV infection, and a small proportion (19.2%) demonstrated poor knowledge. For HPV vaccination knowledge, only 9.7% of the participants demonstrated good knowledge, while the majority (57.0%) exhibited moderate knowledge. Regarding overall HPV infection and vaccination knowledge, a total of 13.9% of the participants had good knowledge, close to one-third (27.6%) had poor knowledge, and 58.5% had moderate knowledge (Fig. 1).

### HPV vaccination practices

Only 13.4% of the participants reported being vaccinated against HPV. Among those who were unvaccinated (86.6%), 80.9% of the participants expressed willingness to receive the HPV vaccine. Only 19.1% of the participants indicated unwillingness, citing barriers such as lack of information about the HPV vaccine (46.0%), safety concerns (46.0%), perceived low risk of infection (12.7%), and religious reasons (9.5%). Seven participants (11.1%) additionally reported unwillingness due to either not having symptoms or being too old (Table 4).

**Table 4:** Human papillomavirus (HPV) vaccination practice among female students at the University of the Free State

Variable		*n* (%)
Vaccinated against HPV (*n* = 381)	Yes	51 (13.4%)
	No	330 (86.6%)
Willingness to receive HPV vaccination (if not vaccinated) (*n* = 330)	Yes	267 (80.9%)
	No	63 (19.1%)
Reasons for unwillingness to receive HPV vaccination (if not willing) (*n* = 63) (multiple choices allowed)	The HPV vaccine is ineffective	2 (3.2%)
	I have no information about the HPV vaccine	29 (46.0%)
	I do not consider myself at risk of HPV infection	8 (12.7%)
	Fear of needle injection	2 (3.2%)
	Safety concern	29 (46.0%)
	Religious reasons	6 (9.5%)
	Other^a^	7 (11.1%)

^a^Includes: I do not have symptoms, I am way above age/too old; I am interested, but I have heard that I am too old. I do not think the vaccine was available when I was younger, and I only heard about it after I was above the age to receive the vaccine

### HPV vaccination across different age groups

The largest proportion (10.8%) of vaccinated participants were in the age group 18–26 (Supplementary Table 2).

### HPV self-sampling awareness and perceptions

Most (78.2%) of the participants were unaware of HPV self-sampling, with only 21.8% of the participants having heard of it (Table 5). A vast majority (86.4%) of the participants had no concerns regarding HPV self-sampling. Of the participants who had concerns (13.6%), these included accuracy and quality of samples (42.3%), procedure concerns (23.1%), and lack of education and information (13.5%).

**Table 5:** HPV self-sampling awareness and perceptions among female students at the University of the Free State

Variable		*n* (%)
Have you heard about HPV self-sampling? (*n* = 381)	Yes	83 (21.8%)
	No	298 (78.2%)
Do you have any potential concerns regarding HPV self-sampling? (*n* = 381)	Yes	52 (13.6%)
	No	329 (86.4%)
If yes, please specify your concerns (*n* = 52)	Accuracy and quality of samples	22 (42.3%)
	Procedure concerns	12 (23.1%)
	Education and information	7 (13.5%)
	Cost and Accessibility	1 (1.9%)
	Health Impact	3 (5.8%)
	General Inquiry	5 (9.6%)
	Missing	2 (3.8%)

### Quoted concerns from participants in response to open-ended questions

#### Accuracy and quality of samples

Participants expressed concern about improper self-sampling, which may lead to poor sample quality.

“Accuracy and low self-efficacy in collection leading to poor sample quality”—P36.

“Improper use leading to false negatives”—P42.

“False-negative results due to individuals not collecting the sample properly”—P303.

### Self-sampling procedure concerns

Participants indicated comfort and pain as their concerns; furthermore, concerns regarding how being a virgin with an intact hymen would impact self-sampling were also raised.

“While I’m open to the idea, I have some concerns about the procedure, particularly for individuals who are virgins and have an intact hymen. The attachment provided by the survey indicates that participants would need to insert a cervical brush into their vagina for the self-sample. This raises questions about how individuals with an intact hymen would navigate this process. As a virgin with an intact hymen, I’m uncertain about the feasibility and potential discomfort associated with inserting the cervical brush. While I understand that the procedure is designed to be minimally invasive and that the hymen is a thin membrane rather than a solid barrier, I still have reservations”—P60.

“I’m concerned about pain”—P177.

“My concerns are related to comfort and pain”—P263.

### Education and information

Some participants had not heard about HPV before partaking in this study, and others suggested that without information and education, the interpretation of results would be inaccurate.

“Without the proper education, the interpretation of the results will not be accurate”—P12.

“I haven’t even heard of HPV before this study; I don’t know what self-sampling is. is it safe, painless and accurate?” P – 179.

### Cost and accessibility

Participants expressed concern about the financial costs of self-sampling.

“It may not be as cost-effective (transport, the swab, paying the lab)”—P16.

### Health impact

Participants expressed concerns related to the health impact of self-sampling, showing a lack of information about HPV self-sampling.

“What effects will self-sampling have on my overall health…?” P64.

### Factors associated with HPV knowledge

Supplementary Table 3 presents the findings from both univariate and multivariable analyses, with overall HPV knowledge as the dependent variable. Participants aged under 26 years (OR: 0.363, 95% CI: 0.043–2.912) were less likely to have comprehensive HPV knowledge compared to those aged 27 years and older (OR: 0.454, 95% CI: 0.052–3.955). Students with a master’s degree (OR: 1.010, 95% CI: 0.190–5.377) showed higher odds of possessing HPV knowledge compared to honors students (OR: 0.617, 95% CI: 0.120–3.186). Additionally, those vaccinated against HPV (OR: 1.915, 95% CI: 0.897–4.086) had increased odds of HPV knowledge. In the univariate analysis, prior awareness of HPV self-sampling (having heard about HPV self-sampling (OR: 2.684, 95% CI: 1.380–5.188, *p* = 0.003)) was a statistically significant predictor of HPV. When adjusted for age, postgraduate level, and awareness of HPV self-sampling in the multivariable regression model, no significant associations were found.

## Discussion

This study evaluated knowledge of human papillomavirus (HPV) infection and vaccination, vaccination practices, and awareness and perceptions of HPV self-sampling among female university students in Free State, South Africa. The findings showed that while 40.9% of participants had good knowledge of HPV infection, only 9.7% demonstrated good knowledge of HPV vaccination, and 13.3% had good overall knowledge, indicating significant gaps in understanding preventive measures among university students. Despite knowledge gaps, most participants were willing to receive the HPV vaccine. Barriers to vaccination included insufficient information, safety concerns, and a perceived low risk of infection. Awareness of HPV self-sampling was limited, with some participants expressing concerns about its accuracy and the procedure relating to discomfort and pain.

Cronbach’s alpha was used to assess the reliability of the questionnaire’s internal consistency [23]. The instrument used in this study exhibits good internal consistency. In the current study, 79.3% of participants were aged 18–26, reflecting a young demographic. The study also highlighted sexual behavior trends, with 73.8% of participants reporting sexual activity and contraceptive use (72.2%), though only 36.0% used condoms, which act as a barrier to sexually transmitted infections (STIs) such as HIV and HPV [24]. The low condom usage is concerning, given South Africa’s high prevalence of STIs [25]. Additionally, 20.2% of participants reported multiple sexual partners, a known risk factor for HPV infection [26, 27]. While only 5.2% reported a history of STIs, this could reflect underreporting or lack of awareness, aligning with prior findings of a high STI burden among young South African women [8, 28–30].

In the current study, 92.7% of participants correctly identified HPV as a sexually transmitted infection. However, awareness was lower in studies among university students from South Africa (66.5%), Ethiopia (48.1%), and Morocco (1.5%) [18, 31, 32]. Approximately 90% of participants in this study knew HPV causes cervical cancer, 71.4% knew it causes genital warts, and 59.3% were aware that HPV often has no symptoms. Similar to the current study, 93.2% of participants in a previous South African study knew that HPV could cause cervical cancer [33], and only 46.6% of students in Ethiopia were aware of its potential to cause genital warts [31]. In contrast, lower awareness rates on the role of HPV in cervical cancer were observed in studies from Ethiopia (46.6%) and Morocco (47.9%) [31, 32]. Comparable to our finding (71.4%), 79.0% of university students in a study conducted in South Africa were unaware of the asymptomatic nature of HPV [15]. A high proportion of participants (88.5%) recognized condoms as protective against HPV, higher than figures reported in South Africa (58.2%) and Ethiopia (28.2%) [18, 31]. HIV-positive women have a higher risk of HPV infection [34]. However, in the current study, only half (50.7%) of the participants knew that HPV infection is higher in HIV-positive women.

The primary objective of HPV vaccination is to prevent invasive cervical cancer by targeting and preventing infection with oncogenic/high-risk HPV types [35]. In South Africa, the vaccine was approved in 2008, and a public school-based program began in 2014 [11]. Awareness of the availability of the HPV vaccine among participants (90.3%) in the current study exceeded figures from studies conducted among South African university students in KwaZulu-Natal (26.2%), Free State (73.4%), and Gauteng (3.2%) [15, 18, 33]. A study conducted among university students in Algeria also reported a lower figure (28.2%) compared to the current study [36]. Additionally, a study conducted among hospital attendees in KwaZulu-Natal found that 12.0% of the participants from urban areas and 7.0% from rural areas were aware of the HPV vaccine [37].

In the current study, 80.6% of the participants were unaware that HPV vaccination is a primary rather than a secondary preventive measure of HPV. More than two-thirds (69.3%) of participants knew that the HPV vaccine could prevent genital warts. This finding is higher than the 45.5% reported in a previous study conducted in Ethiopia [38]. In the current study, over half (56.2%) of participants were unaware that the HPV vaccine prevents new HPV infections but does not eliminate existing ones. HPV vaccination can prevent vaccine-targeted HPV types, and screening can eventually eradicate cervical cancer; however, vaccinated women should still undergo screening [39]. In the current study, 37.3% of participants were unaware that women should still be screened for cervical cancer even when vaccinated. Although 66.7% knew that there is a school-based vaccination program in South Africa, 36.2% were unaware of the recommended ages of vaccination, 56.9% were unaware of the doses that are given, and 96.9% were unaware of the World Health Organization (WHO) Cervical Cancer Elimination Initiative; 90% of girls fully vaccinated against HPV by age 15. Overall, only 9.7% of participants in this study had good knowledge of HPV vaccination. Previous studies in South Africa and Algeria have also reported limited knowledge of the HPV vaccine among university students [33, 36]. Variations in study design, sample size, and timing of the studies may explain some of the differences observed between the studies. Additionally, the level of knowledge might differ between the general population, non-students, and undergraduate and postgraduate students, suggesting that educational background and academic level might influence awareness and knowledge of HPV infection and vaccination.

Vaccination rates were low in this study, with only 13.4% of participants vaccinated. The low vaccination rate observed in the study could be attributed to missed opportunities for vaccination during the initial rollout of the program for those who were within the age range. Various barriers, including safety concerns and vaccine hesitancy, may also have contributed. Similarly, only 5.8% of Algerian university students and 0.9% of Nigerian women aged 25 years and older reported being vaccinated [36, 40]. The low vaccination rate in these studies could be due to HPV vaccination not being part of the vaccination program and not being approved in Algeria [36], and the recent introduction of the vaccine in 2023 in Nigeria [41].

Among unvaccinated participants, 19.1% were unwilling to receive the vaccine, citing safety concerns (46.0%), lack of information (46.0%), perceived low risk of infection (12.7%), and doubts about effectiveness (3.2%). Addressing vaccine hesitancy is crucial to improving public health outcomes. A previous study has reported a slightly higher figure (37.5%) of vaccine hesitancy among students in Algeria [36]. Similarly, vaccine reluctance was linked to not considering oneself at risk (30.4%), concerns about the vaccine’s safety (7.2%), and doubts about its effectiveness (3.2%) [36]. A systematic review in sub-Saharan Africa reported stigma, fear and costs of vaccines, negative experiences with vaccinations, and lack of correct information as barriers to HPV vaccination [42]. Accurate information should be provided about the safety and effectiveness of the HPV vaccine. This includes addressing common myths and misconceptions about the vaccine. Additionally, as expected, the highest proportion (10.8%) of participants vaccinated against HPV in this study were in the 18–26 age group. This age group is most likely to have received the vaccine during their primary school years, approximately 10 years ago, when HPV vaccination programs were implemented in public schools.

South Africa has a high burden of HPV and cervical cancer [2]. Rural areas and underserved populations face significant challenges in accessing regular screening services. Therefore, self-sampling represents a promising tool for increasing coverage in several settings, especially harder-to-reach (rural) populations [16]. In this study, awareness of HPV self-sampling was low, with 78.2% of the participants unaware of the method. A previous study among university students in KwaZulu-Natal reported similar findings, with 95.8% of participants being unaware of HPV self-sampling [15]. Although the participants in the current study were provided with an information sheet about HPV self-sampling, only 13.6% reported having concerns regarding this method. Participants’ concerns included accuracy and quality of the sample (42.3%), procedure concerns such as discomfort and pain (23.1%), and lack of education and information (13.5%). It is important to raise awareness and address concerns about accuracy and procedural comfort to improve self-sampling knowledge and acceptance.

The analysis of overall HPV knowledge stratified by characteristics of the participants provided insights into factors influencing HPV knowledge. Participants enrolled in a Master’s degree program, using contraceptives, had visited a health facility in the past six months and were vaccinated against HPV demonstrated higher odds of having HPV knowledge. However, the results were not statistically significant. A statistically significant association was observed among those who had heard about HPV self-sampling (OR: 2.684, 95% CI: 1.389–5.188, *p* = 0.003). Contraceptive users and those who visited a healthcare facility could be more likely to have greater engagement with reproductive health services, offering more opportunities to learn about HPV. The same applies to vaccinated participants, who might have received health education as part of the vaccination process. The study population, consisting of university students, may show relatively uniform demographic and behavioral characteristics; for example, similar educational environments and access to information might limit the variability needed to detect associations.

## Strengths and limitations

The strength of this study is the use of open-ended questions on concerns about HPV self-sampling and unwillingness to receive the HPV vaccine. This added valuable context and enriched the findings by capturing participants’ specific perspectives. Additionally, the feedback mechanism incorporated into the survey provided correct answers immediately after completion and ensured that participants gained knowledge, aligning with the study’s goal of promoting health education. While this study yielded significant findings, it is important to acknowledge its limitations. The reliance on self-reported data could have introduced response bias. Since the study population was composed of only university students, the findings may not be generalizable to all South African university students, as differences in educational levels could influence the results, and the non-response rate was not accounted for in the sample size calculation.

## Conclusion

In conclusion, 40.9% of female university students in Bloemfontein, South Africa, demonstrated good knowledge about HPV infection, only 9.7% had good knowledge about HPV vaccination, and 13.3% demonstrated good overall knowledge across both topics. Despite these levels of understanding, significant gaps remain, particularly regarding HPV vaccination. While most participants expressed willingness to receive the vaccine, barriers such as lack of information, safety concerns, and a perceived low risk of infection persist. Limited awareness of HPV self-sampling highlights the need for targeted education and outreach programs to improve understanding of its role in cervical cancer prevention. Integrating targeted HPV education within university health promotion programs could bridge knowledge gaps and increase vaccine uptake. These initiatives must emphasize the benefits of HPV vaccination and screening while addressing common misconceptions and safety concerns. Furthermore, policymakers could consider expanding school-based HPV vaccination programs to include university students who may have missed vaccination during adolescence. By addressing these gaps, public health efforts can significantly contribute to achieving the ‘90–70–90’ targets of the WHO Cervical Cancer Elimination in South Africa.

## Supplementary Information

Below is the link to the electronic supplementary material.

Supplementary file1 (PDF 884 kb)

Supplementary file2 (DOCX 13 kb)

Supplementary file3 (DOCX 14 kb)

Supplementary file4 (DOCX 18 kb)

## Data Availability

The data that support the findings of this study are available from the corresponding author upon request.

## Abbreviations

HPV: Human papillomavirus
Hr: High-risk
IQR: Interquartile range
LMICs: Low- and middle-income countries
Lr: Low-risk
REDCap: Research Electronic Data Capture
STIs: Sexually transmitted infections
