# Supplementary material for: Knowledge of human papillomavirus and self-sampling, including vaccination practices among female students in Free State, South Africa
Source: Cancer Causes Control. 2025 Aug 23;36(12):1705–17. doi: 10.1007/s10552-025-02049-5 (PMC12630182; doi:10.1007/s10552-025-02049-5)
Supplement: Supplementary file 1 — Supplementary file1 (PDF 884 kb) [file 10552_2025_2049_MOESM1_ESM.pdf]

World Health Organization

human reproduction programme hrp
research for impact
UNDP · UNFPA · UNICEF · WHO · WORLD BANK

WHO recommendations on self-care interventions

Human papillomavirus (HPV) self-sampling as part of cervical cancer screening and treatment, 2022 update

What is self-care?

WHO's definition of self-care is the ability of individuals, families and communities to promote health, prevent disease, maintain health, and cope with illness and disability with or without the support of a health worker.

What are self-care interventions?

Self-care interventions are evidence-based, quality medicines, devices, diagnostics and/or digital products which can be provided fully or partially outside of formal health services and can be used with or without the direct supervision of health care personnel.

WHO guideline on self-care interventions for health and well-being

- There is an estimated shortage of 18 million health workers by 2030, mainly in low-middle income countries.
- At least 400 million people worldwide lack access to the most essential health services.
- During humanitarian emergencies, including pandemics, routine health services are disrupted and existing health systems can be over-stretched.

For certain health services, incorporating self-care interventions can be an innovative strategy to strengthen primary health care, improve universal health coverage (UHC) and help ensure continuity of health services which may otherwise be disrupted due to health emergencies. In 2022, WHO revised the global normative guidance on self-care interventions for health and well-being, with each recommendation based on extensive consultations and a review of existing evidence.

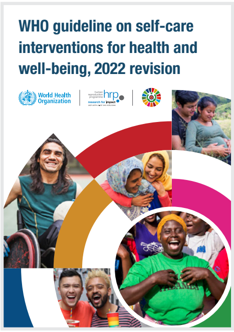

WHO guideline on self-care interventions for health and well-being, 2022 revision
https://www.who.int/publications/i/item/9789240052192

Cervical cancer prevention and screening

- 99% of cervical cancer cases are linked to infection with 'high-risk' types of human papillomavirus (HPV), which is sexually transmitted.
- Vaccination against specific high-risk types of HPV is an important way to prevent cervical cancer; however, women in many countries are not able to access HPV vaccines.
- When diagnosed early and managed effectively, cervical cancer is one of the most successfully treatable forms of cancer.
- Cervical cancer screening and treatment for pre-cancerous lesions is essential to prevent cervical cancer.
- WHO recommends using HPV DNA detection as the primary screening test rather than visual inspection with acetic acid (VIA) or cytology in screen-and-treat approaches among both the general population of women and women living with HIV.

About 90% of new cervical cancer cases and deaths worldwide in 2020 occurred in low-and middle-income countries.*

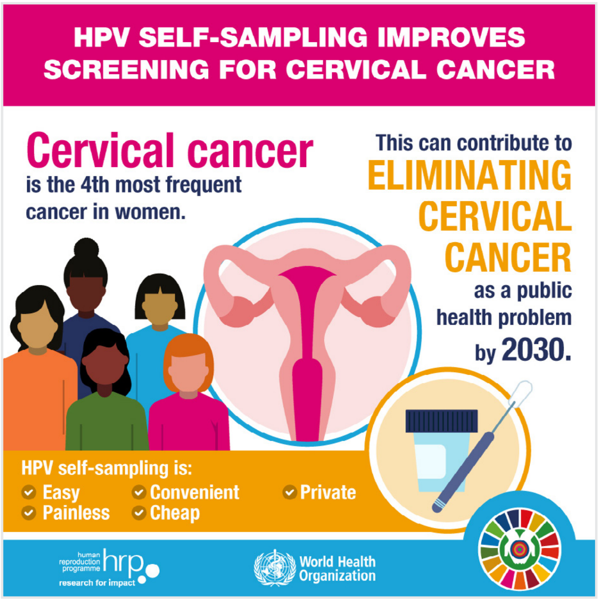

Cervical cancer infographic https://www.who.int/multi-media/details/hpv-self-sampling-improves-screening-for-cervical-cancer

Globally, cervical cancer is the fourth most common type of cancer among women. In 2020, an estimated:

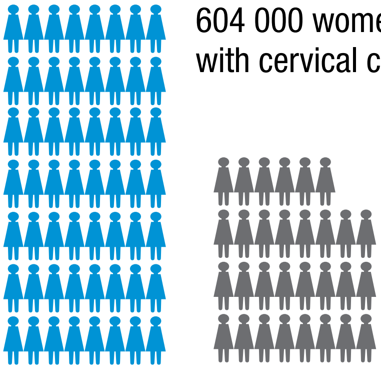

604 000 women were diagnosed with cervical cancer worldwide*

About 342 000 women died from the disease.*

* Source: Ferlay J, Ervik M, Lam F, Colombet M, Mery L, Piñeros M, Znaor A, Soerjomataram I, Bray F (2020). Global Cancer Observatory: Cancer Today. Lyon, France: International Agency for Research on Cancer. Available from: https://gco.iarc.fr/today, accessed 27 January 2023.

Learn more:

Cervical screening and treatment guidelines
https://www.who.int/publications/i/item/9789240030824

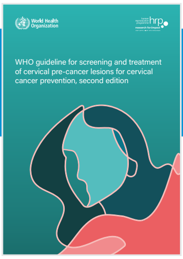

Current challenges to health systems to screen for cervical cancer

- Socio-economic inequalities and health disparities limit access to cervical cancer screening and treatment, which in turn leads to high burden of cervical cancer in low- and middle-income countries.
- In many countries, a majority of women do not have access to cervical cancer screening and treatment services. Women aged 30 and above need to be screened regularly, as pre-cancerous lesions can take many years to develop. For some groups, including women living with HIV, screening should be done starting at age 25.
- In addition to lack of access, other barriers include fear or shame, cultural or religious considerations, distance and cost of travel to services, and time spent for cervical cancer screening and treatment.

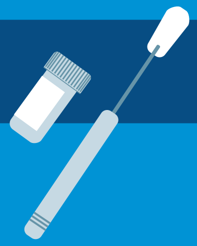

WHO recommends that HPV self-sampling should be made available as an additional approach to sampling in cervical cancer screening services, for women aged 30-60 years.

How does HPV self-sampling work as part of cervical cancer screening and treatment?

Self-sampling involves an individual obtaining a kit and collecting one's own vaginal sample. Collection can be done alone in private, in a health facility or another location. The individual (or a health worker) sends it to a laboratory for testing and the results of the test are returned to the individual. In the case of a positive test result, the individual is linked to follow-up clinical assessments and treatment.

While HPV self-sampling kits may use different methods for sampling and collection, one of the most common methods involves using a single use swab or cervical brush with a tube containing collection/transport medium.

Where HPV tests are available as part of the national programme, the choice to be able to self-sample may encourage women to access screening and treatment services and also improve screening coverage.

Self-sampling can help reach the global target of 70% coverage of screening by 2030. Women may feel more comfortable taking their own samples, rather than going to see a health worker for cervical cancer screening.

Self-collection of a sample for cervical cancer screening by swabbing the vagina[1]

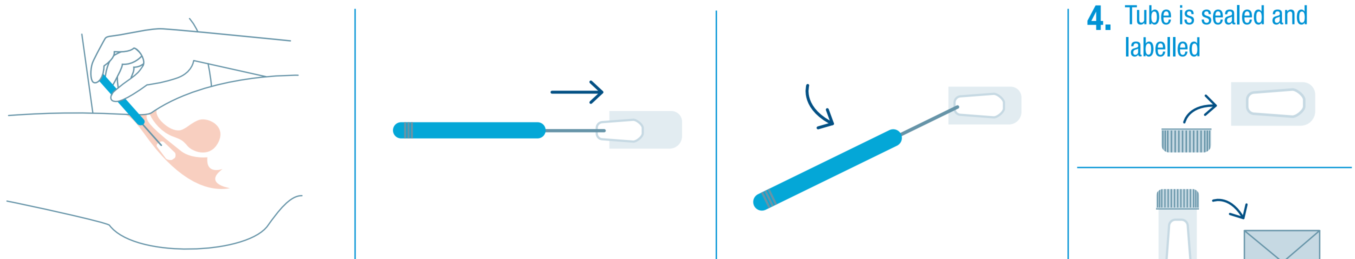

1. The swab or brush is inserted into the vagina and gently rotated for 10-30 seconds
2. The swab or brush is removed and transferred to a provided collection tube
3. The shaft of the swab or brush is broken off and discarded
4. Tube is sealed and labelled
5. Sample is sent to the laboratory via the health worker/clinic/postal services

Effective and acceptable - what the evidence tells us so far

- The option to self-sample is generally associated with increased uptake of cervical cancer screening services: self-sampling nearly doubled use of cervical cancer screening services.
- Self-sampling is seen as highly acceptable for its privacy, convenience, time and effort saved, cost-effectiveness, ease, comfort (including decreased embarrassment, pain and anxiety), speed, safety and user-friendliness.
- Linkages to follow-up testing and treatment after self-sampling and after regular screening remains limited.

[1] The self-collection process may vary by product, but generally follows these steps. Diagram adapted from the WHO technical guidance and specifications of medical devices for screening and treatment of precancerous lesions in the prevention of cervical cancer https://apps.who.int/iris/bitstream/handle/10665/331698/9789240002630-eng.pdf

Considerations for success for HPV self-sampling

- **Information** – Women must be provided with clear information on the benefits of cervical screening and treatment and of self-sampling, as well as detailed information on how to correctly take a sample, and what should be done with the sample.
- **Linkage to follow-up care** – Whether samples are collected by health workers or individuals themselves, a range of evidence based strategies should be used to to facilitate follow-up testing and treatment after self-sampling or screening.
- **Quality products** – Relevant regulatory agencies should ensure that appropriate, quality products are available in adequate quantities. Specifically, regulatory agencies and kit manufacturers should ensure that self-sampling kits are validated for the HPV laboratory tests available.
- **Policy and regulatory frameworks** – Existing national cervical cancer screening policies and strategies should be adapted, developed and/or harmonized to consider HPV self-sampling.
- **Monitoring implementation** – The incorporation of self-sampling into cervical cancer screening systems should be monitored for uptake, use as intended, cost incurred by users, and to identify any related social harm.

Enabling access to the HPV self-sampling kits

Where HPV tests are available, programmes should consider whether the inclusion of HPV self-sampling as a complementary option within their existing approaches to cervical screening and treatment could address gaps in current coverage.

Countries should consider including HPV testing into their national guidelines for cervical cancer prevention, and ensuring that appropriate laboratory infrastructure is in place to process tests. Requests for HPV self-sampling kits can be made by health workers or individuals themselves.

Learn more:

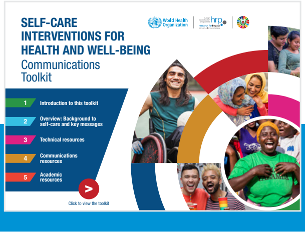

Self-care interventions communications toolkit https://cdn.who.int/media/docs/default-source/reproductive-health/who-selfcare-comms-kit.pdf?sfvrsn=45461bca_3

References:

WHO guideline on self-care interventions for health and well-being, 2022 revision
https://www.who.int/publications/i/item/9789240052192

Self-sampling for human papillomavirus (HPV) testing: A systematic review and meta-analysis
https://gh.bmj.com/content/bmjgh/4/3/e001351.full.pdf

Human papillomavirus (HPV) and cervical cancer Key Facts – WHO website
https://www.who.int/news-room/fact-sheets/detail/human-papillomavirus-(hpv)-and-cervical-cancer

Cervical cancer overview – WHO website
https://www.who.int/health-topics/cervical-cancer

Fact sheet: Self-care health interventions
https://www.who.int/news-room/fact-sheets/detail/self-care-health-interventions

WHO infographic on self-sampling for cervical cancer screening
https://www.who.int/multi-media/details/hpv-self-sampling-improves-screening-for-cervical-cancer

Global strategy to accelerate the elimination of cervical cancer as a public health problem
https://www.who.int/publications/i/item/9789240014107

WHO guideline for screening and treatment of cervical pre-cancer lesions for cervical cancer prevention, second edition
https://apps.who.int/iris/handle/10665/342365

WHO guideline for screening and treatment of cervical pre-cancer lesions for cervical cancer prevention: use of mRNA tests for human papillomavirus (HPV)
https://apps.who.int/iris/handle/10665/350652

Human papillomavirus (HPV) nucleic acid amplification tests (NAATs) to screen for cervical pre-cancer lesions and prevent cervical cancer: policy brief
https://apps.who.int/iris/handle/10665/352495

WHO/SRH/23.1 – © WHO 2023.
Some rights reserved.
This work is available under the CC BY-NC-SA 3.0 IGO licence.
