# Supplementary material for: Knowledge of human papillomavirus and self-sampling, including vaccination practices among female students in Free State, South Africa
Source: Cancer Causes Control. 2025 Aug 23;36(12):1705–17. doi: 10.1007/s10552-025-02049-5 (PMC12630182; doi:10.1007/s10552-025-02049-5)
Supplement: Supplementary file 2 — Supplementary file2 (DOCX 13 kb) [file 10552_2025_2049_MOESM2_ESM.docx]

**Supplementary Table 1:** Reliability Statistics.

| **Statistics** | **Value** |
| --- | --- |
| Cronbach’s Alpha | 0.860 |
| Cronbach’s Alpha based on Standardised Items. | 0.860 |
| Number of items | 20 |
