# Supplementary material for: Knowledge of human papillomavirus and self-sampling, including vaccination practices among female students in Free State, South Africa
Source: Cancer Causes Control. 2025 Aug 23;36(12):1705–17. doi: 10.1007/s10552-025-02049-5 (PMC12630182; doi:10.1007/s10552-025-02049-5)
Supplement: Supplementary file 3 — Supplementary file3 (DOCX 14 kb) [file 10552_2025_2049_MOESM3_ESM.docx]

**Supplementary Table 2:** HPV vaccination by age category.

| **Age category** | **Vaccinated against HPV** | |
| --- | --- | --- |
|  | Vaccinated | Unvaccinated |
| 18-26 | 41 (10.8%) | 261 (68.5%) |
| 27-45 | 9 (2.4%) | 62 (16.3%) |
| ≥46 | 1 (0.3%) | 7 (1.8%) |
