# Supplementary material for: Knowledge of human papillomavirus and self-sampling, including vaccination practices among female students in Free State, South Africa
Source: Cancer Causes Control. 2025 Aug 23;36(12):1705–17. doi: 10.1007/s10552-025-02049-5 (PMC12630182; doi:10.1007/s10552-025-02049-5)
Supplement: Supplementary file 4 — Supplementary file4 (DOCX 18 kb) [file 10552_2025_2049_MOESM4_ESM.docx]

**Supplementary Table 3:** Logistic regression analysis of factors associated with knowledge of human papillomavirus (HPV) among female students

at the University of the Free State.

| **Variable** | **Univariate analysis** | | | | **Multivariable analysis** | | | |
| --- | --- | --- | --- | --- | --- | --- | --- | --- |
|  | OR | 95% Wald Confidence Limits | | p-value | AOR | 95% Wald Confidence Limits | | p-value |
| **Age** | | | | | | | | |
| <26 | 0.363 | 0.043 | 2.912 | 0.335 | 0.473 | 0.051 | 4.347 | 0.508 |
| 27-45 | 0.454 | 0.052 | 3.955 | 0.475 | 0.374 | 0.041 | 3.446 | 0.385 |
| ≥46 | 1 |  |  |  | 1 |  |  |  |
| **Educational level** | | | | | | | | |
| Undergraduates | 0.897 | 0.570 | 1.414 | 0.640 |  |  |  |  |
| Postgraduates | 1 |  |  |  |  |  |  |  |
| **Postgraduate level** |  | | | | | | | |
| Honours | 0.617 | 0.120 | 3.186 | 0.564 | 0.734 | 0.116 | 4.641 | 0.742 |
| Masters | 1.010 | 0.190 | 5.377 | 0.991 | 1.319 | 0.232 | 7.516 | 0.755 |
| Doctorate | 1 |  |  |  | 1 |  |  |  |
| **In a sexual relationship** | | | | | | | | |
| Yes | 0.838 | 0.497 | 1.411 | 0.505 |  |  |  |  |
| No | 1 |  |  |  |  |  |  |  |
| **Contraceptive use** | | | | | | | | |
| Yes | 1.131 | 0.607 | 2.106 | 0.698 |  |  |  |  |
| No | 1 |  |  |  |  |  |  |  |
| **Number of current sexual partners** | | | | | | | | |
| 1 | 2.516 | 0.346 | 18.271 | 0.362 |  |  |  |  |
| 2 | 2.124 | 0.274 | 16.470 | 0.471 |  |  |  |  |
| 3 | 1 |  |  |  |  |  |  |  |
| **History of STIs in the past 6 months** | | | | | | | | |
| Yes | 0.444 | 0.179 | 1.105 | 0.08 |  |  |  |  |
| No | 1 |  |  |  |  |  |  |  |
| **Visited a health facility in the past 6 months** | | | | | | | | |
| Yes | 1.029 | 0.654 | 1.618 | 0.903 |  |  |  |  |
| No | 1 |  |  |  |  |  |  |  |
| **Vaccinated against HPV** | | | | | | | | |
| Yes | 1.915 | 0.897 | 4.086 | 0.093 |  |  |  |  |
| No | 1 |  |  |  |  |  |  |  |
| **Heard about HPV self-sampling** | | | | | | | | |
| Yes | 2.684 | 1.389 | 5.188 | **0.003** | 0.482 | 0.191 | 1.217 | 0.123 |
| No | 1 |  |  |  | 1 |  |  |  |
